# Population-based *FMR1* carrier screening among reproductive women

**DOI:** 10.1007/s10815-024-03242-2

**Published:** 2024-09-25

**Authors:** Quratul Ain, Ye Hyun Hwang, Daryl Yeung, Pacharee Panpaprai, Wiwat Iamurairat, Wiboon Chutimongkonkul, Objoon Trachoo, Flora Tassone, Poonnada Jiraanont

**Affiliations:** 1 Department of Biochemistry and Molecular Medicine, School of Medicine, University of California Davis, Davis, CA USA; 2 Department of Obstetrics and Gynecology, Medical Service Department, Sirindhorn Hospital, Bangkok, Thailand; 3 Department of Medicine, Faculty of Medicine, Ramathibodi Hospital, Mahidol University, Bangkok, Thailand; 4 College of Health Sciences, VinUniversity, Hanoi, Vietnam; 5 UC Davis MIND Institute, University of California Davis, Sacramento, CA USA; 6 Faculty of Medicine, King Mongkut’s Institute of Technology Ladkrabang, Bangkok, Thailand

**Keywords:** FXPOI, FXPAC, Premutation, Carrier screening, Prevalence

## Abstract

**Purpose:**

Fragile X syndrome (FXS) is a neurodevelopmental disorder, caused by an CGG repeat expansion (FM, > 200 CGG) in the fragile X messenger ribonucleoprotein 1 (*FMR1*) gene. Female carriers of a premutation (PM; 55–200 CGG) can transmit the PM allele, which, depending on the CGG allele size, can expand to an allele in the FM range in the offspring.

**Methods:**

Carrier screening for *FMR1* PM is not available in Thailand. This study aimed to investigate the prevalence of PM carriers among Thai reproductive women at the tertiary hospital. A total of 1250 females participated in this study; ages ranged from 20 to 45 years, mean of 30 years (S.D. = 6.27).

**Results:**

Two carriers of a premutation allele, with 32,62 and 32,69 CGG repeats respectively, were identified. This corresponds to 1 in 600 women or 0.17% of the population. Further, three women carrying a gray zone allele (45–54 CGG repeats) were identified (29,51; 29,49; and 30,47 CGG repeats) which equals to 1:400 women or 0.25% of the population. No FM case was detected.

**Conclusions:**

This study heightens the importance of PM carrier screening of women of reproductive age, particularly for the higher risk of developing fragile X–associated primary ovarian insufficiency (FXPOI). Early identification of PM carrier status enhances family planning and fecundity alternatives and improves reproductive health outcomes leading to a better life.

## Introduction

Expansion of the CGG trinucleotide sequence within the 5′ UTR of the fragile X messenger ribonucleoprotein 1 (*FMR1*) gene is implicated in a spectrum of disorders, including fragile X syndrome (FXS) and the fragile X premutation–associated conditions (FXPAC).

While individuals harboring alleles with greater than 200 CGG repeats have the full mutation (FM) causing Fragile X syndrome, individuals harboring the premutation (PM), delineated by having a 55 to 200 CGG repeats allele, are at risk for various conditions, falling under the umbrella of FXPAC. These include fragile X–associated tremor/ataxia syndrome (FXTAS), fragile X–associated primary ovarian insufficiency (FXPOI) and fragile X-associated neurodevelopmental disorders (FXAND) [1].

Approximately 40% of men and 6–18% of women are at risk of developing FXTAS which presents with clinical features including intentional tremors, gait ataxia, parkinsonism, neuropathy, and autonomic dysfunction [2–4]. Further, approximately 20–30% of women carriers of a PM allele, compared to 1% in the general population [5], can develop FXPOI, which entails early menopause, elevating the risks of infertility and hormonal imbalances leading to symptoms like hot flashes and osteoporosis [6–8]. Carrier of a PM can also be affected by FXAND, which is characterized by elevated rates of mental health issues including anxiety and depression [1]. Finally, carriers of a PM also face heightened risks of various medical conditions such as thyroid disorders, fibromyalgia, autoimmune diseases, headaches, and sleep disturbances [1, 9, 10]. In female carriers of a PM, the incidence of immune-mediated disorders may escalate in individuals affected by FXTAS and/or FXPOI, potentially because of the additional effect of these conditions rather than the PM alleles itself [11].

Approximately 1 in 110–250 women are carriers of the *FMR1* PM, impacting over a million women in the U.S. [12–15]. Many become aware of their carrier status through a family history of FXS, while approximately 15–30% are diagnosed due to FXPOI symptoms [16, 17]. Twenty to thirty percent of women with PM experience ovarian function decline, before the age of 40, and, thus, develop FXPOI which is characterized by irregular or absent menstrual cycles, disrupted ovulation, and hormonal imbalances.

Several studies investigated the prevalence of FXPOI found that although *FMR1* CGG repeat and the AGG interruptions do not correlate with age at amenorrhea [18], a nonlinear association between the number of CGG repeats and the ovarian phenotypes has been reported, with FXPOI risk escalating with increasing repeat counts before plateauing or even declining after reaching medium-sized PM alleles of approximately 80–100 CGG repeats [5, 19, 20]. In general, all carrier groups showed a higher prevalence of FXPOI compared to non-carriers, with the medium-sized PM group exhibiting the strongest positive correlation. Similarly, women carriers of a PM experienced a decrease in mean menopausal age, with the medium-sized group showing the lowest mean age of menopause. Further, they enter menopause on average about 5 years earlier than non-carriers. Additionally, menstrual cycle patterns differed among carrier groups, with those in the low-sized (59–79 CGG repeats) and medium-sized PM categories more likely to report shorter cycles and longer intervals between periods; however, the medium-sized group was more prone to irregular cycles. Further, women with medium-sized repeats had lower fertility rates and an increased incidence of dizygotic twinning compared to both non-carriers and other carrier groups. These comprehensive examinations shed light on the varied reproductive health outcomes associated with different CGG repeat allele sizes particularly for the medium-sized group who demonstrated a higher risk for FXPOI and the poorest reproductive health [6, 20–23].

The mechanisms behind compromised ovarian follicular function before the full development of FXPOI are not understood, but it is suggested that these issues may arise at various stages of follicular development with an increase in atresia among the population of growing follicles at all developmental stages [24, 25]. The exact reasons behind the observed early depletion of the ovarian reserve (the pool of non-growing follicles) are not well understood. However, research shows that there are clear interactions between the pools of growing and non-growing follicles that help regulate when follicle growth is activated [26–28]. Despite this knowledge, the specific molecular mechanisms causing FXPOI remain unclear, although there are indications of an RNA toxic effect as the genetic underlined cause [23].

Various prevalence studies conducted in different countries have mainly focused on at-risk neurodevelopmental populations including intellectual disabilities and autism spectrum disorders. However, several studies have investigated allele frequencies in the general population. Among the Asian general population, a few studies [29, 30] have found a significant number of women carrying the PM allele including a large screening study involving 20,188 pregnant Taiwanese women, where 26 carriers of a premutation were detected, which transmitted the expanded allele to 17 fetuses (56.6%), resulting in 6 FM cases. The authors concluded the prevalence of PM in low-risk Taiwanese women is 1 in 777 (0.13%) which was considered to be high, cost-effective, and feasible for carrier screening in Taiwan [29]. In a screening study focused on carrier status conducted in Korea, 8 out of 8641 pregnant women were identified as PM carriers, indicating a prevalence rate of approximately 0.09% within the sampled population [31]. Another screening study in Korea, including 5829 women of reproductive age, identified 7 PMs among 5470 low-risk women, corresponding to a carrier frequency of 1 in 781 (0.13%), rather high among the Asian population [30]. The prevalence of PM carriers among Pakistani preconception women was found to be 6 in 808 (0.7%), 0.5% for women with a family history of ID, and 0.2% for those with a family history of ASD [32]. A study in Turkey found a prevalence rate of 90 out of 263 (34.2%) females harboring the *FMR1* PM and only 0.2% of women had FXPOI [33]. In Spain, a study revealed that 19 out of 84 women (22.6%) were identified as PM carriers at risk for FXPOI [34]. In Israel, pre-conceptional and prenatal screening for Fragile X syndrome detected 231 carriers out of 36,483 women, representing a prevalence rate of approximately 0.63% [35].

These studies emphasize the importance of carrier screening in women before conception. Identification of PM carriers allows tailored counseling and management strategies, ensuring individuals to understand the potential risks to offspring and can make informed choices about family planning.

Here, we report an investigational study in Thailand to screen preconceptionally women of reproductive age, for the presence of the *FMR1* PM. Our goal was to determine the prevalence of the PM carrier in this low-risk population and to understand the experiences of the identified women with a PM before and after receiving the *FMR1* PM diagnosis. By understanding these experiences, healthcare providers can improve care for individuals with FXPOI and offer personalized care and support tailored to the unique needs and preferences of everyone, ultimately promoting their reproductive health and overall well-being.

## Materials and methods

### Subjects

Blood samples were collected from a group comprising 1250 women, whose ages spanned between 20 and 45 years of age and who sought medical attention at the drop-in clinic situated within the Department of Obstetrics and Gynecology at Sirindhorn Hospital, Thailand. This comprehensive study underwent rigorous ethical scrutiny and was granted approval by two esteemed regulatory bodies: the Ethics Committee of King Mongkut’s Institute of Technology Ladkrabang (approval reference number EC_KMITL_63_056) and the Bangkok Metropolitan Administration Human Research Ethics Committee (approval reference number E006h/62_EXP). Each participant voluntarily provided written informed consent, signifying their conscious decision to take part in this project.

The inclusion criteria were thoroughly defined, ensuring a homogeneous study population. Specifically, the study targeted Thai women who were not currently pregnant but expressed aspirations of conception. Furthermore, participants were screened to exclude any clinical evidence of single gene disorders, thereby ensuring the integrity and homogeneity of the group. Additionally, individuals with a history of recurrent pregnancy loss were excluded to maintain the study’s focus and minimize confounding variables.

This approach to participant selection underscores the study’s commitment to scientific rigor and ensures the reliability and validity of the findings derived from this esteemed group. Through adherence to stringent ethical standards and meticulous attention to detail, this study aims to contribute invaluable insights into the intricate interplay of genetic factors influencing reproductive health among Thai women.

### CGG repeat allele sizing

Blood samples from 1250 female participants were collected on the Whatman 903 Protein Saver Card (Buckinghamshire, UK). The blood spot cards were allowed to dry and stored at room temperature until ready for the genotyping analysis. DNA isolation was carried out from 2 × 1.2 mm blood spots (Whatman 903 Protein Saver Card) according to the manufacturer’s instructions. The Eppendorf tubes containing the washed blood spots were boiled for 10 min in a boiling water bath and subsequently centrifuged at 13,000 rpm to eliminate any remaining liquid before transferring into a clean PCR reaction tube and subjected to PCR. Amplification of the *FMR1* gene was performed using a combination of the Asuragen AmplideX® PCR/CE *FMR1* Kit (Asuragen, Austin, TX, USA) using the CGG trinucleotide primer and the FastStart Taq DNA Polymerase kit by Millipore Sigma Aldrich (Roche Diagnostics, Mannheim, Germany) according to the manufacturer instructions.

A Genetic Analyzer, ABI 3130 XL, was utilized for the determination of PCR product sizes. Subsequent analysis was conducted using Peak Scanner software (version 2.0; Thermo Fisher Scientific) in accordance with the guidelines provided by the manufacturer. Alleles were classified into distinct categories including full mutation (> 200 CGG repeats), premutation (55–200 CGG repeats), intermediate (45–54 CGG repeats), and normal (< 44 CGG repeats).

## Results

There were 1250 females who participated in this study. Participants’ age ranged from 20 to 45 with the majority (64%), being 20–30 years old, obtained bachelor’s degree (43%), and have low income between 10,000–30,000 Baht per month (57%). Common medical problems seeking treatment/intervention included allergy and asthma, major depressive disorder and anxiety disorders, thyroid dysfunction, diabetes, hypertension, dyslipidemia, migraine, thalassemia, and anemia, respectively.

**Fig. 1:**
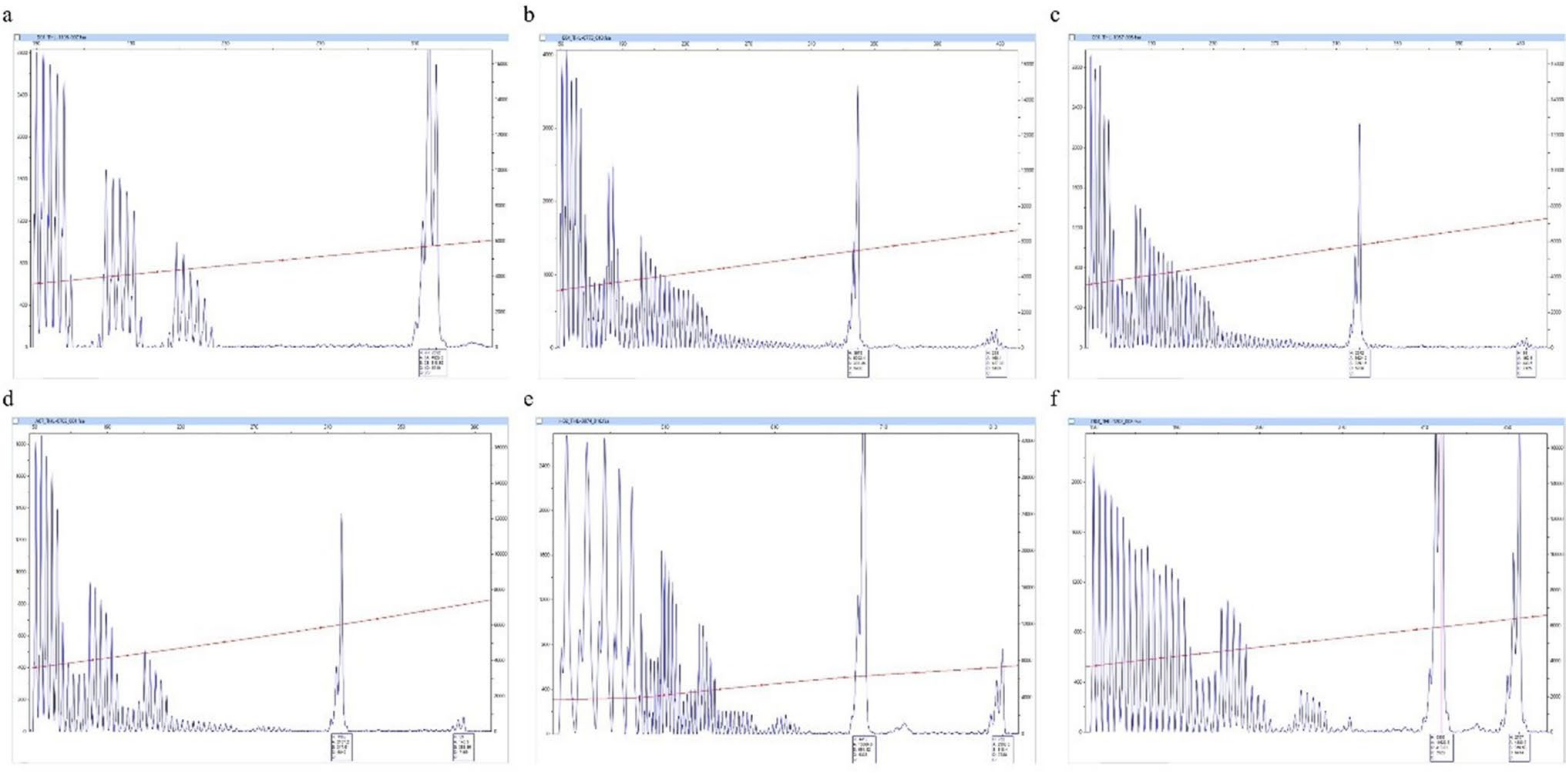
CGG plots of women with **a** normal allele, **b**, **c** premutation alleles, and **d**, **e**, **f** gray zone alleles

A total of 1245 women (99.6%) harbored an *FMR1* allele within the normal CGG repeat range (Fig. 1a). No cases of FM were detected among participants. Two women (0.17% of the total population) presented an allele in the PM range, of 32,62 and 32,69 CGG repeat length with no AGG interruptions (Fig. 1b and c). One female reported having migraine. Further, three women (0.25% of the total group) had a gray zone allele, associated with intermediate CGG repeat lengths. Their CGG repeat numbers were 29,51, 29,49, and 30,47 respectively, with one or two AGG interruptions (Fig. 1d, e, f). They reported no medical problems.

## Discussion

To our knowledge, this is the first screening that investigated the prevalence of PM carriers among the Thai female population. Of 1250 female participants, two carriers of a PM were identified. No FM case was detected which is not surprising given the small cohort studied and since the ratio of FM in women was reported to be 1:11,000 (Hunter et.al., 2014). Comparing our findings with population-based studies examining the prevalence of *FMR1* PM carriers among preconception and pregnant women in diverse populations reveals intriguing insights. Notably, the prevalence rates observed in our study align with or fall below those reported in other ethnic and geographic groups. For instance, prevalence rates in Korean, Chinese, Australian, Israeli, Pakistani, and Spanish women vary from 0.04 to 1.3%, indicating significant variability across populations [29, 30, 32, 36–38]. Among the Asian population, our PM prevalence is relatively higher than that of Taiwanese and Korean studies at 1:777 and 1:781, respectively, although our samples are much smaller than these two large studies. It is possible that increasing the sample size may find a higher ratio of PM carriers among the Thai female population.

Significantly, women carrying PM alleles face an elevated risk of having affected children due to the expansion of the repeat to the FM (> 200 CGG repeats), a phenomenon that occurs when the mutation is passed from mother to child [39]. However, the identification of PM carriers highlights the need for comprehensive reproductive counseling and support services. Women identified as carriers of the PM allele may benefit from fertility evaluations, hormone assessments, and genetic counseling to understand their risk of developing FXPOI and explore reproductive options. Recommendations from various medical organizations advocate for *FMR1* testing for all women exhibiting unexplained ovarian insufficiency or elevated follicle-stimulating hormone (FSH) levels before the age of 40, irrespective of family history [16, 19, 40, 41]. Enhanced clinician awareness of FXPOI is imperative for timely diagnosis and follow-up care to mitigate medical risks and improve quality of life [42, 43]. Younger women who develop FXPOI are expected to face a prolonged period of uncertainty before receiving a diagnosis [16], a delay attributed to healthcare providers’ limited understanding of FXPOI and to the rarity of the condition, which necessitates patients to advocate for themselves more actively [12, 44].

Moreover, molecular analysis identified both PM and gray zone alleles in our study. The presence of one or two AGG interruptions within the CGG repeats of gray zone alleles was demonstrated, highlighting the intricate genetic landscape underlying FXPOI susceptibility. Both the number of CGG repeats and the presence of AGG triplets within the CGG repeat segment play a significant role in determining the likelihood of expansion [45, 46] and in gray and small PM alleles containing 45–69 repeats, a clear link between the number of AGG interruptions, the length of uninterrupted CGG repeats and maternal age has been reported. These factors have been associated with the instability of maternal alleles and the subsequent risk of repeat expansion when transmitted to offspring [47, 48]. While our study’s sample size may have been limited, our findings corroborate these previous findings, highlighting the importance of assessing repeat stability, particularly for women with alleles falling within the gray zone. The presence of alleles within the gray zone raises important considerations regarding their potential implications on reproductive health and ovarian function, warranting further investigation into their phenotypic consequences.

In women of reproductive age, being a carrier of the PM allele often goes unnoticed unless clinicians may diagnose PM-carrier women based on a family history of Fragile X–related disorders. While the PM carrier status typically remains silent, it signifies a genetic predisposition that may elevate the risk of Fragile X–associated conditions, albeit to a lesser extent than the FM [49].

The research underscores the impact of knowing one’s premutation carrier status on reproductive choices, given the risk of having an FXS-affected child and the heightened infertility risk associated with FXPOI [6, 12, 50]. Women with the PM and/or FXPOI symptoms should receive comprehensive reproductive counseling during their childbearing years, encompassing fertility evaluations, hormone assessments, genetic counseling, and guidance on reproductive options such as conceiving naturally, assisted reproductive technologies, or opting for adoption [23, 51]. Thus, these findings further emphasize the importance of comprehensive genetic screening programs to identify at-risk individuals and facilitate timely interventions and support. By identifying PM carriers early, healthcare providers can offer initiative-taking management and support to mitigate the risk of FXPOI-related complications.

Despite the valuable insights provided by this study, several limitations should be acknowledged. The relatively small sample size and localized nature of the study population limit the generalizability of the findings to broader populations of Thai women. Future research could aim to replicate these findings in larger groups and explore additional factors contributing to FXPOI risk, such as environmental influences and genetic modifiers.

Longitudinal studies tracking the reproductive outcomes and health trajectories of PM carriers are also needed to assess the long-term implications of *FMR1* PMs on ovarian function and overall well-being. By monitoring individuals over time, researchers can gain insights into the progression of FXPOI and the efficacy of interventions aimed at preserving fertility and improving quality of life for affected individuals.

## Conclusion

In conclusion, this investigation emphasizes the importance of genetic screening for *FMR1* PM alleles in women from the general population. By identifying PM carriers early and providing personalized reproductive counseling and support, healthcare providers can empower women to make informed decisions about their fertility options and family planning strategies, improving reproductive health outcomes and quality of life.

## Data Availability

The data that support the findings of this study are available on request from the corresponding author, [PJ]. The data are not publicly available due to privacy/ethical of participants.
